# A Century of Otorhinolaryngology at Yonsei University College of Medicine and Engagement With US Missionaries

**DOI:** 10.1002/lio2.70088

**Published:** 2025-02-10

**Authors:** In Seok Moon, Min‐Seok Rha, In‐Sok Yeo, Chang‐Hoon Kim

**Affiliations:** ^1^ Department of Otorhinolaryngology Yonsei University College of Medicine Seoul Republic of Korea; ^2^ Department of Medical History & Institute for History of Medicine Yonsei University College of Medicine Seoul Republic of Korea

**Keywords:** history, Missionary Medical School, otorhinolaryngology, Severance, Yonsei

## Abstract

**Objective:**

To provide a historical overview of the Department of Otorhinolaryngology at Yonsei University College of Medicine and highlight its contributions to the development of otorhinolaryngology in Korea.

**Data Sources:**

Historical records of the Dongeun Medical Museum, Yonsei University College of Medicine, and related publications.

**Review Methods:**

Historical review.

**Results:**

Gwanghyewon, the first modern hospital established by American missionaries in Korea, later became the Severance Hospital (named after American philanthropist Louis Severance) and Yonsei University College of Medicine. The Department of Otorhinolaryngology at Yonsei University and Severance Hospital was established in 1908. Currently, the department has 24 professors and 6 clinical fellows specializing in otology, rhinology, and laryngology‐head and neck surgery.

**Conclusion:**

In the past 116 years, Yonsei Otorhinolaryngology has been devoted to the great clinical and academic development of otorhinolaryngology in Korea. The next century of Yonsei Otorhinolaryngology will build on the progress made in its legacy.

**Level of Evidence:**

N/A.

## Graphical abstract

This article provides a historical overview of the Department of Otorhinolaryngology at Yonsei University College of Medicine and highlights its contributions to the development of otorhinolaryngology in Korea.

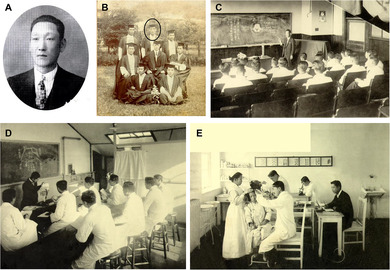

*With the Love of God, Free Humankind from Disease and Suffering*.

The above phrase is the mission of Yonsei University College of Medicine, the first missionary medical school in Korea. Inheriting this philosophy, the Department of Otorhinolaryngology at Yonsei University College of Medicine has achieved significant clinical and academic milestones in the history of otorhinolaryngology in Korea. This paper documents the development and history of Yonsei Otorhinolaryngology. It celebrates more than 115 years of otology, rhinology, and laryngology‐head and neck surgery practice at Severance Hospital, the first modern hospital established by American missionaries in Korea. This report was compiled from various sources, including the historical archives of the Dongeun Medical Museum, the Severance Hospital Reports and Overview (1886–1958), the Severance Union Medical College Graduation Album, annual report documents from the Department of Otorhinolaryngology at Yonsei University College of Medicine, and publications such as the 50th Anniversary History of the Korean Society of Otorhinolaryngology‐Head and Neck Surgery and the 70th Anniversary History of the Korean Society of Otorhinolaryngology‐Head and Neck Surgery. The authors verified and conducted an integrative analysis of records from these diverse sources. To ensure accuracy and reliability, records were carefully selected based on their relevance and credibility. Each source was cross‐referenced with others to identify and minimize potential biases associated with relying solely on institutional archives. Any discrepancies identified during the review were critically examined, and additional efforts were made to corroborate details through alternative records or expert consultations. By employing this methodology, we aimed to present a comprehensive and reliable account of Yonsei Otorhinolaryngology's historical contributions and legacy. This article is organized into three sections: (1) the birth of Severance Hospital and Yonsei University College of Medicine, (2) the history of the Department of Otorhinolaryngology at Yonsei University College of Medicine, and (3) the current status of the department.

## 1 The Birth of Severance Hospital and Yonsei University College of Medicine

**FIGURE 1:**
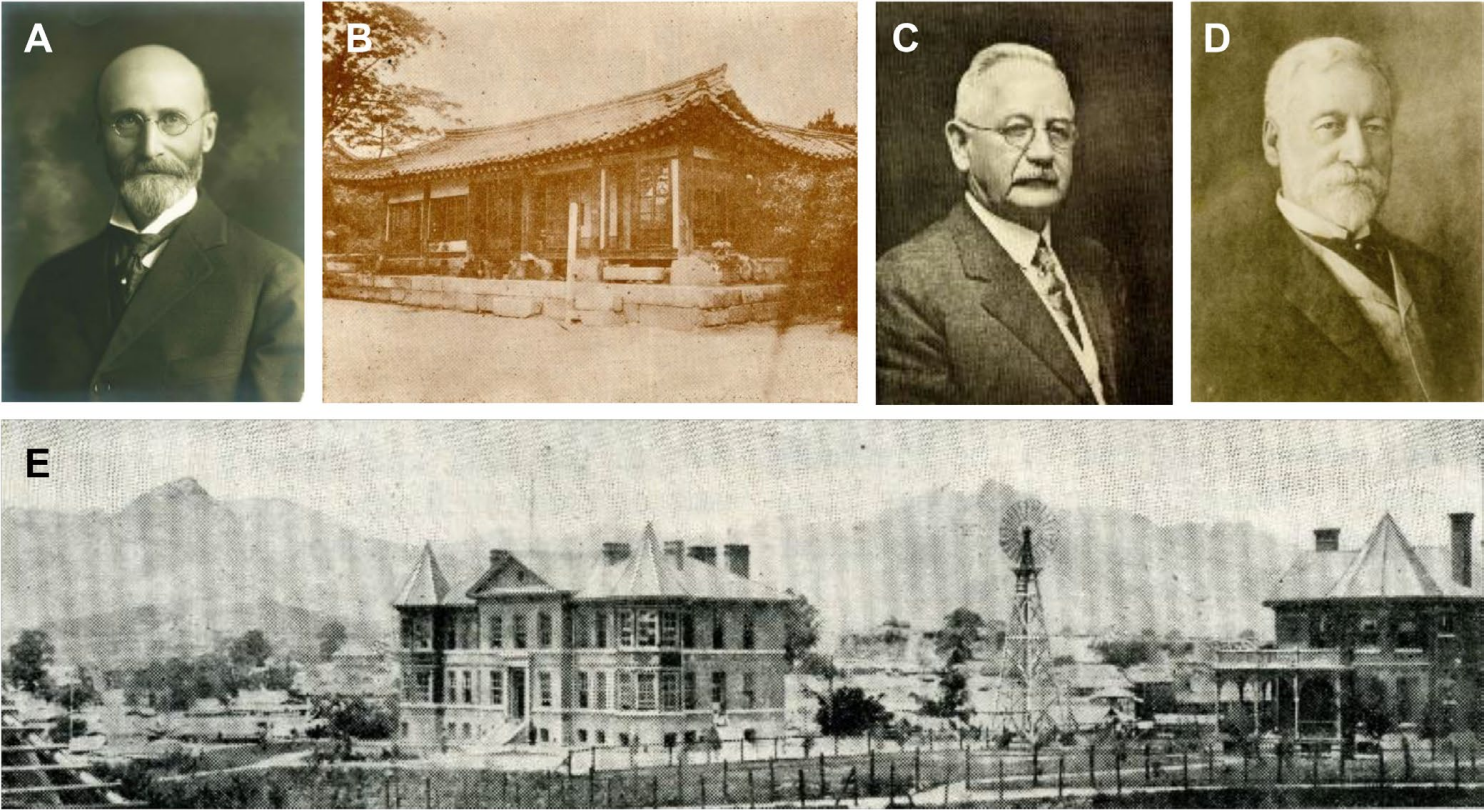
The beginning of Severance Hospital and Yonsei University College of Medicine. (A) Horace Newton Allen, an American Presbyterian missionary who introduced modern medical treatment to Korea through Chejungwon. (B) The establishment of Gwanghyewon in 1885. (C) Oliver R. Avison, a Canadian missionary and physician who served as the General Director of Jejungwon. (D) Louis Henry Severance, an American philanthropist who made a significant financial contribution to build a new Hospital. (E) An early panoramic photograph of Severance Hospital. These images were obtained from the Dong‐Eun Medical Museum.

In 1884, Horace Newton Allen (Figure 1A), an American missionary physician from a Presbyterian mission in New York, treated Queen Myeongseong's nephew Young‐ik Min, who was severely injured. Experts specializing in Chinese Oriental medicine, including herbal medications, acupuncture, and moxibustion, were in charge of public health in Korea. Young‐Ik Min recovered completely within 3 months under the care of Horace Allen. King Gojong was impressed by the modern medicine practiced by Horace Allen and consequently supported the establishment of Gwanghyewon in 1885 (Figure 1B) as the first modern hospital in Korea. In the same year, the hospital was renamed Chejungwon, and in 1886, it became Chejungwon Medical School, Korea's first Western medical school. Oliver R. Avison (Figure 1C), a missionary doctor originally from the University of Toronto, became Chejungwon's fourth director in 1893. For 50 years, Avison devoted himself to developing modern medical practices and establishing training systems in South Korea. On September 3, 1904, the hospital was renamed Severance Hospital in honor of American philanthropist Louis Henry Severance (Figure 1D,E). In 1908, the first seven graduates received a doctoral license from the Korean government. The role of traditional medicine has gradually decreased since the introduction of Western medicine. During the Japanese occupation, public health was rapidly modernized by the government and missionaries. Japan eliminated oriental medicine from the public health system and built Western‐style hospitals to care for Japanese people residing in Korea. Missionary doctors actively spread their knowledge of Western medicine among Koreans as a means of religious work. In 1962, the Severance Hospital evolved into the current Yonsei University health system. The system consists of three colleges (Medicine, Dentistry, and Nursing) and four hospitals (Severance Hospital, Dental Hospital, Gangnam Severance Hospital, and Yongin Severance Hospital).

## 2 The History of Yonsei Otorhinolaryngology

### 2.1 The Beginning and Early History of Otorhinolaryngology at Severance Hospital

**FIGURE 2:**
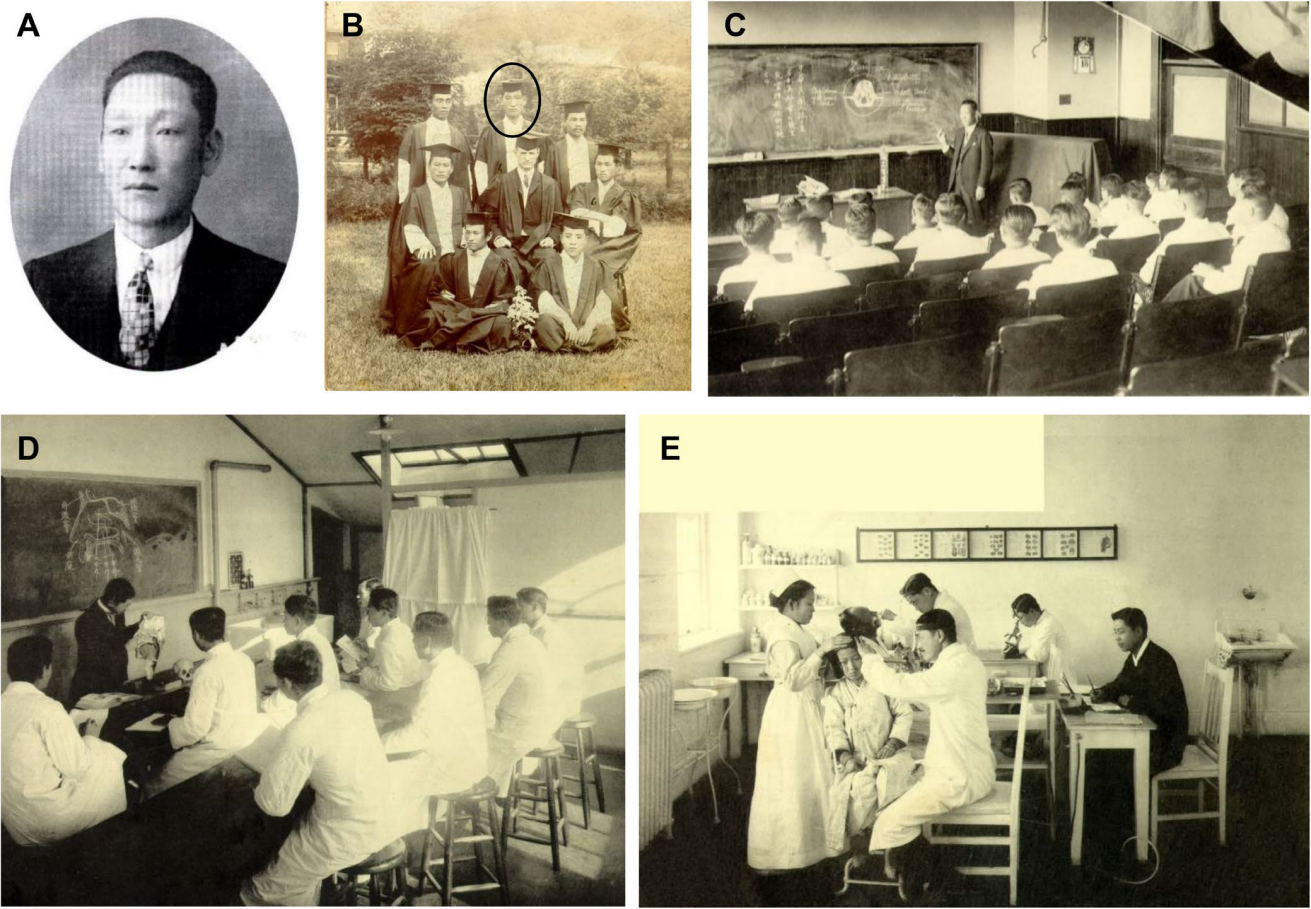
Suk‐Hoo Hong, the first Korean otorhinolaryngologist, and the inception of otorhinolaryngology education in Korea. (A) Doctor Suk‐Hoo Hong. (B) The first seven graduates in Chejungwon Medical School. The black circle indicates Suk‐Hoo Hong. (C) An otorhinolaryngology lecture by Suk‐Hoo Hong. (D) A lecture by Oka Shinobu in 1917. (E) Outpatient clinical practice in otolaryngology led by Suk‐Hoo Hong in 1917. These images were obtained from the Dong‐Eun Medical Museum.

Suk‐Hoo Hong, one of the first seven graduates of Chejungwon Medical School, became the first Korean otorhinolaryngologist and Assistant Professor in 1908 (Figure 2A−C). He initially worked as a specialist in otolaryngology and ophthalmology at Severance Hospital. In 1921, he moved to the United States for training and studied at Kansas City University and City University of New York. After completing his two‐year program, he returned to serve as the chairman of the Department of Otorhinolaryngology. In the early years of Severance Hospital, clinical practice, and training in ophthalmology and otolaryngology were integrated. However, as the clinical fields and skills of these two specialties diverged, otorhinolaryngology was fully separated from ophthalmology in 1917, establishing itself as an independent department. Lectures and clinical training in otorhinolaryngology have been conducted at Severance Union Medical College since its inception (Figure 2D,E).

Following its establishment at the College of Medicine in 1945, Byung‐Hyun Lee was the first chairman of the department. Lee made significant advancements in the field of otolaryngology. He performed the first stapes mobilization surgery in Korea and introduced suspension laryngoscopy. Additionally, he served as the third and sixth chairman of the Korean Society of Otorhinolaryngology‐Head and Neck Surgery and acted as the Dean of Yonsei University College of Medicine from 1962 to 1964, making notable contributions to the development of education.

The second chairman, Gill Ryoung Kim, made significant contributions not only to clinical activities but also to research from 1968 to 1984. He made significant efforts to enhance the department's international presence. In 1977, Kim established Korea's first vocal testing laboratory, the “Vocal Dynamics Laboratory,” and he conducted suspension laryngoscopy, laryngeal stroboscopy, and laser laryngeal microsurgery, playing a pioneering role in the field of voice medicine in Korea. Joining the International Federation of Otolaryngology Societies in 1977, Kim contributed significantly to international society's development and continued his service as an advisor from 1985. He also served as the second president of the Korean Society of Otorhinolaryngology‐Head and Neck Surgery. In 1983, Kim chaired the organizing committee for the 5th Asia‐Oceania Otolaryngology Conference, the first international conference held in Korea, and successfully hosted the event.

As the number of graduates from the department increased, O‐Kong‐Hoe, the alumni association of Yonsei Otorhinolaryngology, was established in 1955. Currently, 489 regular members and 26 junior members are active. The annual activities of O‐Kong‐Hoe include academic activities, such as the O‐Kong academic symposium, the publication of regular bulletins, and various social activities.

In 1983, Yeongdong Severance Hospital (currently Gangnam Severance Hospital) was established under the leadership of Young Myoung Kim in the Gangnam region, an area with limited healthcare at the time. He played a pivotal role in the construction of the hospital and advancing the otolaryngology department within the new healthcare facility.

In 1987, “The Commission for Advancement of Yonsei Otorhinolaryngology” was established to discuss detailed plans for the improvement of diverse aspects of the department, including clinical practice, scientific research, and education. Recognizing the increasing complexity of otolaryngological diseases and the need for specialized knowledge and skills, our department introduced a subspecialty system for the first time in Korea in 1988. The department was divided into three subspecialties: otology, rhinology, and laryngology‐head and neck surgery. This innovative system allowed faculty members to concentrate on their primary expertise.

### 2.2 Major Achievements in the Clinical Field

**FIGURE 3:**
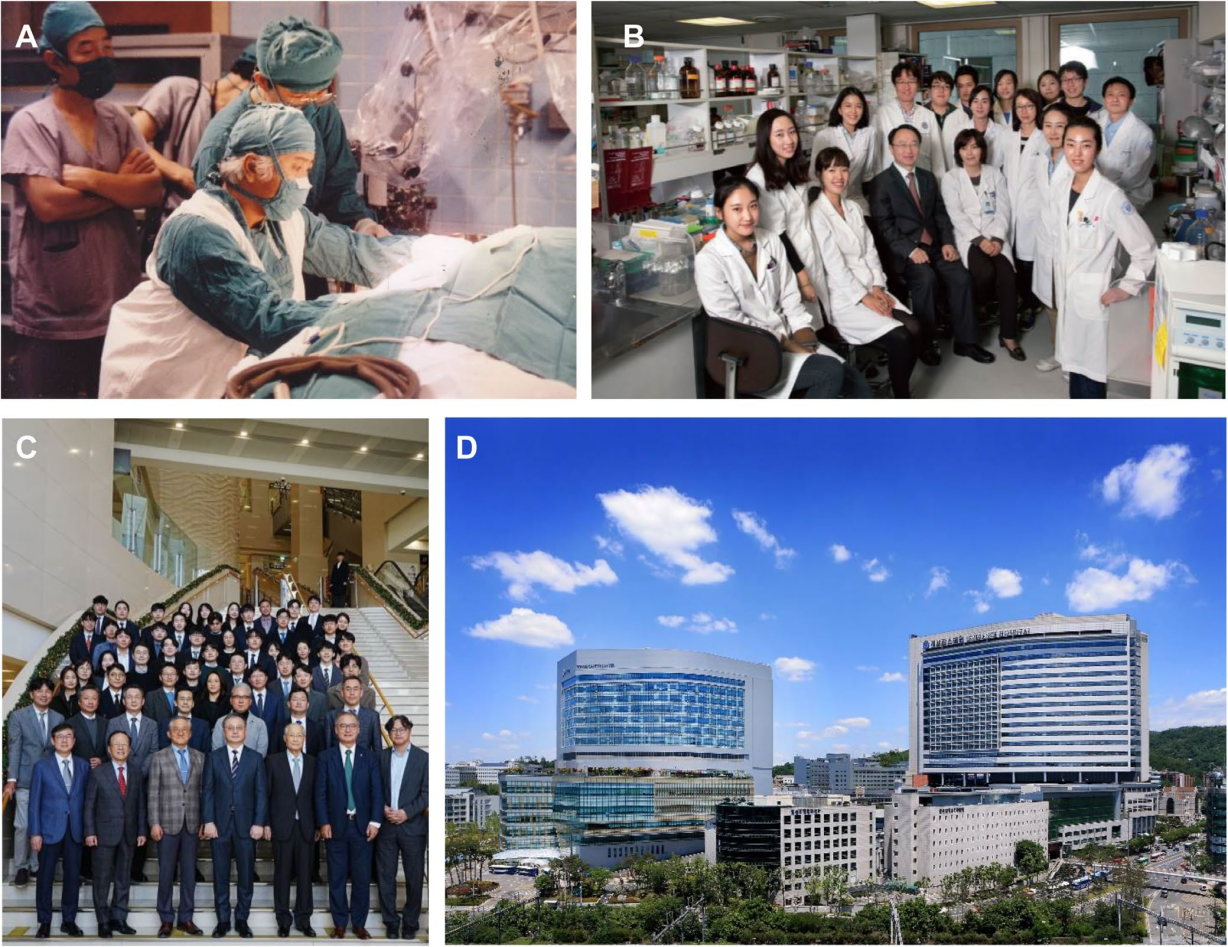
Development and the current status of the department. (A) Photograph illustrating the first cochlear implantation surgery performed in Korea by Hee Nam Kim in 1988. (B) Photograph of the members of the Airway Mucus Institute, established for advanced basic research in the field of otorhinolaryngology in 2004. (C) A photograph of department members inside the Yonsei Cancer Center, Severance Hospital, taken January 6, 2024. Currently, 24 professors, 6 clinical fellows, and 22 residents work in the department. (D) A panoramic view of Severance Hospital taken in 2017.

In Korea, Yonsei Otorhinolaryngology has been at the forefront of advancing modern clinical practice and surgical techniques. The department achieved a significant milestone in 1970 by pioneering microscopic tympanoplasty in Korea. In 1988, Hee Nam Kim introduced tinnitus‐retraining therapy and performed the first cochlear implantation surgery in Korea (Figure 3A). In 2008, Won Sang Lee and Jae Young Choi conducted Korea's first auditory brainstem implantation for a patient with hearing loss. Further expanding their expertise, Jae Young Choi achieved another first in Korea in July 2011 by successfully conducting middle ear implantation.

The history of rhinology in Korea has also been closely intertwined with advancements made in Yonsei Otorhinolaryngology. In 1988, Park introduced an innovative method for endonasal sinus surgery, leveraging an operating microscope and a newly designed self‐retaining retractor speculum, as reported in *Acta Oto‐laryngologica* [1]. In 1993, Jeong Gweon Lee pioneered laser resection of the palatopharynx, marking its first introduction in Korea. Advancing into the new millennium, in 2001, In Yong Park, Jeong Gweon Lee, and Joo‐Heon Yoon conducted an extensive dissection of over 100 cadavers, analyzing the detailed surgical anatomy of the nasal cavity and paranasal sinuses in Koreans. This endeavor led to the discovery of new anatomical findings that contributed to the establishment of a novel surgical concept. The outcomes of this clinical nasal anatomy research were disseminated through numerous peer‐reviewed papers in SCI/SCIE journals and a textbook titled “Surgical Anatomy of the Nose.” In 2019, Joo‐Heon Yoon, Chang‐Hoon Kim, and Hyung‐Ju Cho developed the YSK Yonsei olfactory function (YOF) test kit. This innovative kit, which comprises odors culturally relevant to Koreans, has become a widely used tool in various institutions for evaluating olfactory function and diagnosing patients with olfactory dysfunction.

In 1953, Byung‐Hyun Lee achieved a significant milestone by pioneering laryngeal surgery in the field of head and neck surgery through the introduction of suspension laryngoscopy in Korea. In 1995, Kwang Moon Kim played a pivotal role in advancing treatment options for spasmodic dysphonia with the introduction of Botox injections. In 2008, Se‐Heon Kim made groundbreaking contributions by introducing transoral robotic surgery (TORS) in Asia and performing the world's first TORS for the excision of laryngeal and hypopharyngeal cancers. In 2010, Yoon Woo Koh revolutionized minimally invasive neck surgery by developing a novel technique. He achieved the world's first submandibular gland resection using the retroauricular approach, and further developed neck dissection via the retroauricular approach using the Da Vinci robotic surgical system. By April 2023, more than 3000 patients have benefitted from this innovative surgical technique. In September 2013, Eun Chang Choi and Yoon Woo Koh compiled their technology and expertise in surgical skills, resulting in the publication of the book “Atlas of Head and Neck Surgery: Endoscopic and Robotic Neck Surgery.”

Recognizing his excellence and expertise in head and neck cancer, Eun Chang Choi was honored with an invitation to deliver the Eugene N. Myers International Lecture on Head and Neck Cancer, the honorary guest lecture at the American Academy of Otolarnygology‐Head and Neck Surgery Foundation Annual Meeting, in 2018. During this distinguished lecture, Eun Chang Choi provided in‐depth insights into superselective neck dissection, esthetic head and neck surgery, and metastasis of head and neck cancer.

### 2.3 Pioneering Otolaryngology Research in Korea

The history of research of the Department of Otolaryngology at Yonsei University mirrors the evolution of otolaryngology research in Korea. In 1960, the department made its debut in the scientific community by publishing its first SCI paper in the *Archives of Otolaryngology*. Led by Kim, the groundbreaking research presented a statistical study of 200 cases of benign and malignant tumors in the larynx [2]. This seminal work fueled a growing passion for research within the department, leading to subsequent impactful contributions. In 1987, Kim et al. developed a laryngeal pacemaker in dogs using a temperature sensor, as published in *The Laryngoscope* [3].

During his overseas training at the National Institute of Environmental Health Sciences in the United States from 1995 to 1997, Joo‐Heon Yoon conducted research on respiratory epithelial cell cultures. Upon returning to Korea, he applied this technique to the nasal mucosa, resulting in the development of the world's first primary culture method for nasal epithelial cells in 2000 [4]. This innovative experimental method laid the groundwork for further basic rhinology research. Subsequently, the Airway Mucus Institute was established in 2004 by Joo‐Heon Yoon, with great support from alumni and faculty members (Figure 3B). This institute has conducted cutting‐edge research on respiratory mucosal immunity, hosted an annual symposium to share knowledge with distinguished experts, and has built an international research network. In 2007, the Research Center for Human Natural Defense System, directed by Joo‐Heon Yoon, was selected as a beneficiary of the Korean Government's Science Research Center research fund, which has been implemented since the 1990s to foster groups of leading scientists at the academic level. The Research Center for Human Natural Defense System has received US$1,000,000 of research funding annually for 9 years and has published a total of 230 SCI/SCIE papers.

Currently, the department's three subspecialty sections are engaged in translational and convergence biomedical research. This approach involves an integrative analysis of clinical metadata and experimental data to discover new therapeutic targets and develop novel treatment strategies for otorhinolaryngological diseases. The otology team is dedicated to genomics‐based precision medicine for genetic hearing loss, whereas the rhinology team primarily investigates immune responses in the human nasal mucosa with unprecedented precision. The head and neck team focuses on developing patient‐derived organoids and elucidating the molecular signatures of head and neck cancers using high‐throughput omics technologies.

### 2.4 Yonsei Otorhinolaryngology as a Professional Educational Institution

Since its establishment, Yonsei Otorhinolaryngology has served as a professional training institution in Korea. The mission of our department is centered on “Fostering paramount physicians and scientists to enhance public health.” In addition, one of the three visions of the department is “Developing and inspiring physicians and scientists in otorhinolaryngology.” Recognizing the transformative power of education, we actively engage in training the next generation of otorhinolaryngology leaders, understanding that sharing knowledge through education is pivotal to the evolution of the field. Our emphasis extends beyond local leadership as we aim to nurture the development of national and international leaders in otorhinolaryngology. As of 2024, the department had graduated 217 residents and 113 Doctors of Philosophy. Currently, 79 graduates of Yonsei Otorhinolaryngology serve as faculty members at 28 universities and contribute to healthcare at 31 hospitals. Inspired by Horace Newton Allen, Oliver R. Avison, and Louis Henry Severance, the Yonsei University Health System and Severance Hospital actively participate in educating doctors from developing countries. Annually, more than five visiting scholars from around the world join the Department of Otorhinolaryngology. As of February 2024, 157 foreign scholars had visited the department to gain insight into its clinical and research expertise.

The department has implemented various specialized education programs to train its residents, clinical fellows, and external trainees effectively. In 1997, the Yonsei temporal bone dissection course began to teach essential surgical techniques in neuro‐otologic surgery, including mastoidectomy, tympanoplasty, ossiculoplasty, facial nerve decompression, cochlear implantation, endolymphatic sac surgery, and the translabyrinthine approach. This course attracts not only external trainees from Korea but also international fellows seeking to enhance their otology surgical skills. It is currently held twice a year and approximately 20 attendees complete the course annually. The Yonsei nose cadaver workshop, initiated to provide insights into practical surgical anatomy, involves medial‐to‐lateral dissection of the nasal cavity and paranasal sinuses as well as endoscopic sinus surgery on cadavers. The 42nd workshop was successfully held in 2024. The rhinology team also launched the Yonsei rhinoplasty cadaver workshop and Yonsei sinonasal tumor cadaver workshop to educate participants in the step‐by‐step procedures for rhinoplasty and sinonasal tumor surgery. Similarly, the Yonsei head and neck cadaver workshop, developed by the head and neck team, addresses techniques in head and neck surgery, including procedures such as thyroidectomy, submandibular gland resection, parotidectomy, and neck dissection.

Since 1983, the department has played a crucial role in the academic and social development of otorhinolaryngologists in Seoul through the Continuing Medical Education Program for Primary Care Otorhinolaryngologists. Over the past 40 years, this program has been a central hub for academic activities and social exchange within the community. Serving as a platform for medical education, it enables otolaryngologists in the Seoul region to maintain their academic competence and remain informed about novel clinical skills. These activities are conducted through lectures, written publications, online programs, and other electronic media. The conference celebrated its 40th anniversary in April 2023.

## 3 Current Status of the Department

Currently, 24 professors, 6 clinical fellows, and 22 residents work in the Department of Otorhinolaryngology at Yonsei University College of Medicine (Figure 3C,D). Our faculty members are actively participating in and devoting themselves to clinical practice, research, and education at three hospitals: Severance Hospital, Gangnam Severance Hospital, and Yongin Severance Hospital. The department's outpatient clinics are equipped with 18 examination rooms (10 in Severance Hospital, 4 in Gangnam Severance Hospital, and 4 in Yongin Severance Hospital) and state‐of‐the‐art facilities for nasal function, hearing function, vestibular function, and speech language tests, and speech therapy. In 2023, more than 77,000 outpatients visited our clinics, and over 4000 surgeries were performed. As a result of persistent research efforts, the annual number of SCI/SCIE publications reached 50. In 2023, the total research fund for faculty members in the department was $5,000,000 USD a year.

## 4 Summary

In the past 116 years, Yonsei Otorhinolaryngology has been devoted to the great clinical and academic development of otorhinolaryngology in Korea. As the first otorhinolaryngology department established in Korea, Yonsei Otorhinolaryngology has been a pioneer in advancing modern clinical practices and research within the country. Yonsei's contributions are recognized not only nationally but also internationally, making it one of the world's leading institutions in the field. Compared to other top medical centers, Yonsei has demonstrated remarkable achievements, including performing over 2000 robotic head and neck surgeries—a volume that ranks it among the most experienced institutions globally. Additionally, Yonsei has significantly contributed to the field with numerous publications in high‐impact journals and its leadership in conducting pioneering basic research. Its role in training international scholars further underscores its global influence and distinguishes it from many peer institutions. The next century of Yonsei Otorhinolaryngology will build on the progress made in its legacy. Our department looks forward to training future clinical care and research leaders. More importantly, we are eager to make new discoveries in the field of otorhinolaryngology and translate these into clinical applications.

## Conflicts of Interest

The authors declare no conflicts of interest.
